# Refractory and Super-Refractory Status Epilepticus in Nerve Agent-Poisoned Rats Following Application of Standard Clinical Treatment Guidelines

**DOI:** 10.3389/fnins.2021.732213

**Published:** 2021-09-10

**Authors:** Julia E. Morgan, Sara C. Wilson, Benjamin J. Travis, Kathryn H. Bagri, Kathleen T. Pagarigan, Hannah M. Belski, Cecelia Jackson, Kevin M. Bounader, Jessica M. Coppola, Eden N. Hornung, James E. Johnson, Hilary S. McCarren

**Affiliations:** ^1^Neuroscience Department, US Army Medical Research Institute of Chemical Defense, Aberdeen Proving Ground, MD, United States; ^2^Comparative Pathology Department, US Army Medical Research Institute of Chemical Defense, Aberdeen Proving Ground, MD, United States

**Keywords:** status epilepticus, refractory, seizure, nerve agent, organophosphate, soman

## Abstract

Nerve agents (NAs) induce a severe cholinergic crisis that can lead to status epilepticus (SE). Current guidelines for treatment of NA-induced SE only include prehospital benzodiazepines, which may not fully resolve this life-threatening condition. This study examined the efficacy of general clinical protocols for treatment of SE in the specific context of NA poisoning in adult male rats. Treatment with both intramuscular and intravenous benzodiazepines was entirely insufficient to control SE. Second line intervention with valproate (VPA) initially terminated SE in 35% of rats, but seizures always returned. Phenobarbital (PHB) was more effective, with SE terminating in 56% of rats and 19% of rats remaining seizure-free for at least 24 h. The majority of rats demonstrated refractory SE (RSE) and required treatment with a continuous third-line anesthetic. Both ketamine (KET) and propofol (PRO) led to high levels of mortality, and nearly all rats on these therapies had breakthrough seizure activity, demonstrating super-refractory SE (SRSE). For the small subset of rats in which SE was fully resolved, significant improvements over controls were observed in recovery metrics, behavioral assays, and brain pathology. Together these data suggest that NA-induced SE is particularly severe, but aggressive treatment in the intensive care setting can lead to positive functional outcomes for casualties.

## Introduction

Acts of terrorism and warfare by hostile governments and militant groups have extended beyond the battlefield environment into civilian spaces, often with explicit intent to cause the greatest level of destruction, chaos, and harm possible. Nerve agents (NAs) have been used to execute these types of attacks because they are easily disseminated, fast acting, and highly potent chemical weapons. Developed during World War II, and subsequently banned nearly three decades ago, NAs have continued to make global headlines for their use in targeted political assassinations and mass terrorist attacks (Chai et al., 2017, 2018; Stone, 2018). NAs are organophosphate compounds that cause toxicity by directly inhibiting acetylcholinesterase. This inhibition leads to a prominent, systemic hyper-concentration of the neurotransmitter acetylcholine (ACh) within synapses. Excessive ACh can result in miosis, hypersecretions, fasciculations, pulmonary edema, centrally-induced apnea, and seizures that, if left untreated, can rapidly progress to *status epilepticus* (SE).

*Status epilepticus* is a medical emergency that requires pharmacological intervention to resolve, and increased latency to treatment is associated with a worsened prognosis (Gainza-Lein et al., 2018a,b). This time dependency would become particularly crucial in a mass NA casualty scenario, where treatment would be delayed by physical decontamination procedures, and the number of victims may be far greater than the number of first responders and antiseizure medications available in the field. Current prehospital treatment guidelines for NA poisoning recommend administration of up to 30 mg diazepam (DZP) or 20 mg midazolam (MDZ) to control convulsions in adults (Chemical Hazards Emergency Medical Management (CHEMM), 2021); however, preclinical studies have shown that doses equivalent to 30–120 mg of benzodiazepines do not provide lasting seizure control and are often entirely ineffective against NA-induced SE if administration is delayed more than 5 min (Walton and Treiman, 1988; Mazarati et al., 1998; Rice and DeLorenzo, 1999; Shih et al., 1999; Deshpande et al., 2007; McDonough et al., 2010; Jackson et al., 2019). Thus, it is reasonable to expect that intensive in-hospital care would be required to definitively treat NA-induced SE. There are currently no guidelines for treating NA-induced SE when prehospital benzodiazepines fail, so care would likely follow clinical protocols for SE of non-specific etiology.

Outside of the context of NA exposure, SE can arise from a wide variety of underlying causes that may not be immediately apparent, which can make selecting an effective therapeutic approach extremely difficult. However, there is strong consensus that administration of benzodiazepines [typically MDZ, lorazepam (LRZ), or DZP] within the first 30 min of SE is effective in the majority of cases (Treiman, 1990; Shorvon, 2001; Brophy et al., 2012; Silbergleit et al., 2012). SE that continues or recurs after initial benzodiazepine treatment is categorized as established SE. Common therapeutic choices for established SE include antiepileptic drugs like levetiracetam (LEV), phenobarbital (PHB), phenytoin (PHT), fosphenytoin (FOS), and valproate (VPA). There is little evidence to suggest that any particular drug will be most likely to control SE. In fact, a landmark clinical trial demonstrated that regardless of whether LEV, FOS, or VPA was administered, the efficacy rate against established SE was approximately 50% (Kapur et al., 2019). When antiepileptics fail to terminate seizure activity, the patient is considered to be in refractory SE (RSE). RSE is approached with continuous administration of general anesthetics like ketamine (KET), MDZ, pentobarbital, or propofol (PRO) for 24–48 h at doses that elicit electoencephalographic (EEG) burst suppression. If SE recurs upon weaning from anesthesia, the condition is ultimately classified as super-refractory SE (SRSE).

Given the severity and generalized nature of the cholinergic crisis that results from NA exposure, it is expected that NA-induced SE will rapidly advance to RSE or SRSE (Jagoda and Riggio, 1993; Neligan and Shorvon, 2010; Waheed et al., 2014). It is imperative to control NA-induced SE as quickly as possible, as just 4 h of continuous seizure activity has been sufficient to produce frank neuropathology in non-human primates and rodents (Baze, 1993; McDonough et al., 1998; Jackson et al., 2019). Prolonged SE is associated with a high mortality rate, and the pathological sequelae associated with SE can cause permanent and severe decrements to quality of life. Despite this, there is hope for recovery from NA-induced SE, especially among younger casualties without underlying comorbidities, as long as seizure control can be achieved and systemic NA toxicity can be managed (Alvarez and Drislane, 2016). An intensive care unit (ICU) would be best suited for these challenges because extensive physiological monitoring and supportive interventions like supplemental oxygen and i.v. fluids are available to help manage the primary medical emergency as well as secondary deleterious effects of pharmacological interventions. The work presented here utilized a simulated ICU environment to treat NA-induced SE in rats according to the above-outlined tiered pharmacological approach for clinical SE management. The intent of this project was to determine whether NA-induced SE progresses to a refractory state, and whether particular therapeutics lead to better seizure control at each stage of SE. Establishing a protocol for SE treatment is instrumental in reducing the mortality and morbidity associated with the condition (Shorvon, 2001); thus, identifying a treatment regimen that is specifically adapted to the unique physiological and biochemical environment of NA-induced SE represents the best chance at protecting casualties from lasting harm.

## Materials and Methods

### Ethics Statement

This study was approved by the Institutional Animal Care and Use Committee (IACUC) at the United States Army Medical Research Institute of Chemical Defense, and all procedures were conducted in accordance with the principles stated in the Guide for the Care and Use of Laboratory Animals, and the Animal Welfare Act of 1966 (P.L. 84-544).

### Animals

Male Sprague Dawley rats (*Rattus norvegicus*) between 7 and 8 weeks old and weighing 226–250 g were purchased from Charles River Laboratory (*n* = 304). Rats were pre-implanted with jugular vein catheters by the vendor at least 4 days prior to shipment. Rats were singly housed in polycarbonate cages to prevent damage to catheters and EEG headpieces by cage mates. Except during experiments, they were provided with standard rodent chow and filtered water *ad libitum*. Ambient conditions were maintained in both the housing and experiment rooms at 68–79°F and 30–70% relative humidity. A 12:12-h light-dark cycle with lights on at 0600 was used.

### EEG Surgery

EEG lead implantation surgery was performed 5–6 days after rats arrived at the facility. Analgesia was provided in the form of meloxicam (1 mg/kg, 5 mg/ml in saline) delivered s.c. at least 15 min prior to surgery and again 24 h after surgery. Lidocaine (0.1 ml, 2 mg/ml in saline) was also delivered intradermally at the surgical site just prior to incision. Rats were anesthetized with isoflurane (3–5% induction, 1–3% maintenance in oxygen @ 0.5–1.0 L/min) and placed in a stereotaxic frame. A PhysioSuite unit (Kent Scientific, Torrington, CT, United States) was used to monitor heart rate, SpO_2_, and core body temperature throughout the procedure. Scalp hair was removed, and the surgical site was cleaned with betadine and isopropyl alcohol. Next, the skull was exposed with a midline incision approximately 2–3 cm in length. Burr holes were drilled bilaterally over the parietal cortex and unilaterally over the cerebellum. Stainless steel screw electrodes were placed in the holes and connected to a plug that was fixed in place with glass ionomer dental cement (GC America Inc., Alsip, IL, United States). The skin around the headpiece was closed with 4–0 monofilament simple interrupted sutures. Immediately following removal from the stereotax, rats were administered warm saline (8 ml s.c.), and an RFID temperature transponder (Bio Medic Data Systems, Seaford, DE, United States) was placed s.c. in the opposite flank. The rat was then placed in a heated chamber and continuously monitored until fully recovered. Rats were returned to their home cage with dietary enrichments and observed at least twice a day for the next two days for signs of pain or discomfort.

### Drugs

All drugs were diluted or dissolved in saline unless otherwise noted. HI-6 (Kalexsyn, Kalamazoo, MI, United States) was dissolved at 250 mg/ml. Atropine methyl nitrate (AMN) (Fresenius Kabi, Lake Zurich, IL, United States) was dissolved at 4 mg/ml. Atropine sulfate (Medisca Inc., Irving, TX, United States) was diluted to 1.8 mg/ml and then admixed 1:1 with 2-PAM (ScienceLab Inc., Houston, TX, United States) at 100 mg/ml for prehospital delivery. For repeated in-hospital delivery, atropine sulfate was dissolved at 0.6 mg/ml. MDZ (Hospira, Inc., Lake Forest, IL, United States) was delivered at the manufacturer’s concentration of 5 mg/ml in the prehospital setting and diluted to 2 mg/ml for i.v. delivery in the ICU. LRZ (West-Ward Pharmaceuticals, Memphis, TN, United States) was diluted to 1.2 mg/ml. PHB (West-Ward Pharmaceuticals) was used at the manufacturer’s concentration of 130 mg/ml. VPA (Sigma-Aldrich, St. Louis, MO, United States) was dissolved at 390 mg/ml. KET (Zoetis, Parsippany, NJ, United States) was diluted to 7.5 mg/ml for bolus delivery or 12 mg/ml for continuous delivery. PRO (Ivaoes Animal Health, Miami, FL, United States) was used at the manufacturer’s concentration of 10 mg/ml for both bolus and continuous delivery.

### Nerve Agent Exposure

**FIGURE 1:**
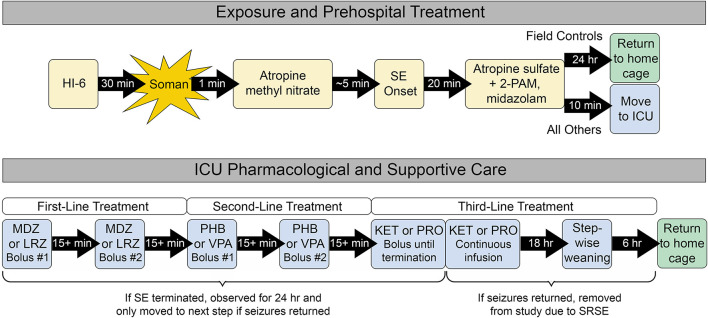
Exposure, prehospital treatment, and ICU care paradigm. All rats received prehospital treatment. Field control rats remained in the prehospital environment for up to 24 h and did not receive further supportive care. ICU controls and ICU-treated rats were transferred to the ICU environment, where they received supplemental oxygen and thermal support. Only the ICU-treated group received additional first-, second-, and third-line pharmacological therapies as needed to control seizure activity. All control rats that survived for 24 h after prehospital treatment and all ICU-treated rats that remained seizure-free for 24 h were returned to their home cage for daily monitoring, behavioral testing 9 days after exposure, and euthanasia 11 days after exposure.

Cohorts of eight rats were placed into individual Plexiglas^®^ chambers and connected to an EEG monitoring system (MP160, BIOPAC Systems Inc., Goleta, CA, United States) to enable real-time display of brain activity using AcqKnowledge^®^ 5.0 software. After approximately 30 min of baseline, NA exposure proceeded as previously described and shown in Figure 1 (Althaus et al., 2017; McCarren et al., 2018; Jackson et al., 2019; Johnstone et al., 2019; Barker et al., 2020). The NA used was soman (150 μg/kg s.c.). Pre-treatment with HI-6 (125 mg/kg i.p.) and post-treatment with AMN (2 mg/kg i.m.) served to protect rats from succumbing to the systemic toxicity of NA exposure before onset of SE. Despite these precautions, 123 rats died prior to receiving therapeutic treatments and were thus excluded from all reported data. Onset of SE was defined as the appearance of repetitive spikes and sharp waves with an amplitude greater than twice that of baseline EEG and having a sustained duration of more than 10 s. In all cases, SE continued unabated for at least the next 20-min or until rats died. Twenty minutes after onset of SE, MDZ (1.8 mg/kg), and atropine sulfate (0.45 mg/kg) admixed with 2-PAM (25 mg/kg) were administered i.m. at doses that represent the recommended prehospital limits for these standard medical countermeasures, which are currently the only approved treatments for NA exposure (Chemical Hazards Emergency Medical Management (CHEMM), 2021). The 20 min treatment time point was chosen because it balances rodent mortality with realistic expectations for first-response time in a real-world NA exposure (Jackson et al., 2019). Within 10 min of receiving standard medical countermeasures, all rats except for the field control group were transferred to individual ThermoCare^®^ ICUs for supportive and pharmacological care. Field control rats remained in the original exposure chamber. All rats received additional doses of atropine sulfate (0.15 mg/kg) i.m. every 3 h for the next 24 h to mitigate systemic NA toxicity.

### Supportive Care

Upon entry into the ICU, rats’ body temperatures and SpO_2_ levels were monitored every 15 min for the first hour and hourly thereafter. Temperature was measured non-invasively using the transponder that was implanted during surgery. Oxygen saturation was measured with a clip placed on the hind foot using the MouseOx Plus pulse oximetry system (STARR Life Sciences^®^ Corporation, Oakmont, PA, United States). The interior of the ICU chambers was kept at 23.7 ± 1.6^*o*^C and 45.2 ± 6.8% humidity (mean ± SD). If a rat’s body temperature exceeded 40°C, the chamber’s warming element was turned off. Supplemental oxygen was delivered to the ICU chamber at 7 L/min, resulting in 12 volume exchanges per hour. Oxygen was delivered for at least the first hour and continued for any rat with a SpO_2_ reading < 94% at the hourly checks.

### Pharmacological Care

Prior to the start of each experiment, rats were assigned to a control group or randomized to receive one of two treatment options at each stage of SE as described in Figure 1. ICU control rats received saline in place of first- and second-line treatment. Treatments were delivered by i.v. bolus through the pre-implanted catheter, followed by ∼0.2 ml saline to ensure that the entire volume of drug was delivered to the bloodstream. Following administration of each drug bolus, rats were evaluated in real time for termination of SE, defined as a stop in repetitive spikes and sharp waves that lasted for at least 5 min. If SE was successfully terminated, rats were monitored for the next 24 h for recurrence of SE or discrete seizures, defined as a bout of spikes or sharp waves with an amplitude at least twice as large as baseline and lasting at least 10 s. Six rats were excluded from all analyses because incorrect treatment decisions were made during the experiment based on post hoc review.

The pharmacological care regimen was designed to mimic clinical guidelines for treatment of SE (Shorvon and Ferlisi, 2011; Glauser et al., 2016). First-line treatment consisted of either MDZ (1 mg/kg) or LRZ (0.6 mg/kg) i.v. If SE was not controlled after the initial bolus, a second bolus was delivered 15 min later. If SE was initially controlled but later returned, a second bolus was delivered at the first sign of returning seizure activity. Any persistence or return of seizure activity within 24 h of the second bolus led to initiation of second-line treatment with PHB (60 mg/kg) or VPA (180 mg/kg) i.v. Similar to first-line treatment, if SE terminated with the first bolus and then returned, a second bolus was administered. However, if the first bolus failed, treatment progressed directly to third-line therapy with KET (4.5 mg/kg) or PRO (6 mg/kg) i.v. KET and PRO boluses were delivered every 15 min until SE terminated. Anesthesia was then maintained for 18 h with continuous infusion of KET (30 mg/kg/h) or PRO (25 mg/kg/h) via programmable syringe pump. This was followed by step-wise tapering of drug each hour for 6 h. If, during any stage of pharmacological care, rats remained seizure-free for 24 h, they were deemed a treatment success and returned to their home cage for continued evaluation. However, seizure activity that returned after therapy progressed through all three lines of treatment was considered SRSE. These rats were euthanized with ≥ 75 mg/kg pentobarbital i.p. followed by bilateral thoracotomy.

### Post-exposure Monitoring and Care

Control rats that survived for 24 h and ICU-treated rats that remained seizure-free for 24 h were returned to their home cage and provided with dietary supplements. All rats received once-daily saline (4 ml) s.c. starting 8 h after NA exposure and lasting for a least 3 days after returning to their home cage. Rats were weighed daily, and saline injections continued until baseline (pre-exposure) weight was achieved or until the end of the study. Each day, rats were scored based on weight change relative to baseline, breathing, natural behavior, and provoked behavior. Each category was scored on a 0- to 3-point scale as detailed in Supplementary Table 1, with lower total scores reflecting better recovery. After the first day, rats with a total score of 6 + were euthanized unless mitigating factors were identified by PI and veterinarian consultation. Three rats met these criteria: two field controls and one ICU control. Additionally, one ICU-treated rat was removed from the study early because its EEG headpiece detached. Data from these four rats were excluded from histopathology and behavioral analysis, but included in all other analyses. These rats were deeply anesthetized with ≥ 75 mg/kg pentobarbital i.p., and a bilateral thoracotomy was performed to confirm euthanasia.

### Behavioral Testing

Two behavioral testing sessions occurred, with the first session three to four days before NA exposure and the second session nine days after NA exposure. Rats were moved to the behavioral testing suite in their home cages and allowed to acclimate to the new room for at least 10 min before testing began. Testing began at 0700-0800. The order in which rats were tested was randomized prior to the start of each session. All rats were tested in the open field test (OFT) first and then in the elevated plus maze (EPM). A single 300-s trial of each test was performed during each testing session. All behavioral data were collected using ANY-maze video tracking software (Stoelting Co., Wood Dale, IL, United States). Equipment was cleaned with 70% ethanol between rats.

For the OFT, rats were placed in the center of a 100 cm × 100 cm field with a solid floor and transparent walls, and they were allowed to freely explore for the duration of the trial. Locomotion was evaluated based on time spent immobile as well as on total distance traveled. Anxiety-related behavior was evaluated based on time spent in the center of the field, which was defined as being at least 20 cm from any of the walls.

The testing apparatus for the EPM consisted of a plus-shaped maze with two closed arms and two open arms that were 10 cm wide × 50 cm long. Walls on the closed arms were 40 cm high, and the entire apparatus was elevated 50 cm from the floor. Rats were initially placed in the center of the field with their nose toward an open arm. Locomotion was again evaluated based on time spent immobile and total distance traveled. Anxiety-related behavior was evaluated based on time spent in the open arms of the field. Exploratory behavior was evaluated based on number of entries into a new arm, which was determined by when the center of the rat’s body crossed into the designated zone.

### Perfusion and Histology

Eleven days after NA exposure, rats were anesthetized with ≥ 75 mg/kg pentobarbital i.p. Each rat was assessed for lack of eye blink reflex and failure to respond to a strong toe pinch prior to opening the chest cavity. Rats were then perfused with 0.9% phosphate buffered saline followed by 10% neutral buffered formalin. Brains were extracted, embedded in paraffin, and sectioned coronally at 5 μm. The section corresponding to 3.24 mm posterior to bregma was stained with H&E according to conventional methods. H&E was scored by a treatment-blinded, board-certified pathologist using a standard scoring system previously utilized to rate the degree of NA-induced brain damage (McDonough et al., 1986, 1995, 1998; McCarren et al., 2020).

### Statistics

Survival, initial SE control, and lasting SE control rates for different treatment conditions were compared using Fisher’s exact tests. Body temperatures and SpO_2_ levels were compared using mixed-effects analyses with Tukey’s multiple comparison tests to compare treatment conditions at each time point. For analysis of long-term outcomes, the specific drug regimen that was used to achieve lasting seizure control was hypothesized to have no effect on any measurement. This was confirmed by comparing all metrics for the group of ICU-treated rats that received MDZ + PHB to the group that received LRZ + PHB. As shown in Supplementary Table 2, there were no differences between these groups. Thus, all of the rats from these two treatment groups, as well as the single rat treated with MDZ + PHB + KET, were collapsed into a single “ICU-treated” group for final comparisons against control groups. Body weights and post-exposure recovery scores were compared using mixed-effects analyses with Tukey’s multiple comparison tests to compare treatment conditions at each time point. Outcomes from behavioral testing were evaluated using 2 × 3 way repeated-measures ANOVAs with Sidak’s multiple comparison tests to compare treatment groups at each testing time point. Histopathology scores in each brain region were compared using Kruskal–Wallis tests with Dunn’s multiple comparison tests.

## Results

### Study Design

Adult male Sprague Dawley rats were implanted with EEG headpieces and jugular vein catheters to allow for visualization of brain activity and i.v. delivery of therapeutic agents throughout the study. SE was induced via s.c. NA exposure. Rats were treated with standard prehospital countermeasures, and then transferred to an ICU environment where they received supplemental oxygen and thermal support along with additional pharmacological interventions according to the tiered approach described in Figure 1. For each stage of treatment, rats were randomly assigned to one of two commonly-utilized therapeutic options. In addition to the rats that received pharmacological interventions, two control groups were included: one group that did not enter the ICU environment and thus did not receive any supportive care (field controls), and a second group that was placed in the ICU environment for supportive care but received saline in place of first-line and second-line treatment (ICU controls).

### Survival and Seizure Outcomes

**FIGURE 2:**
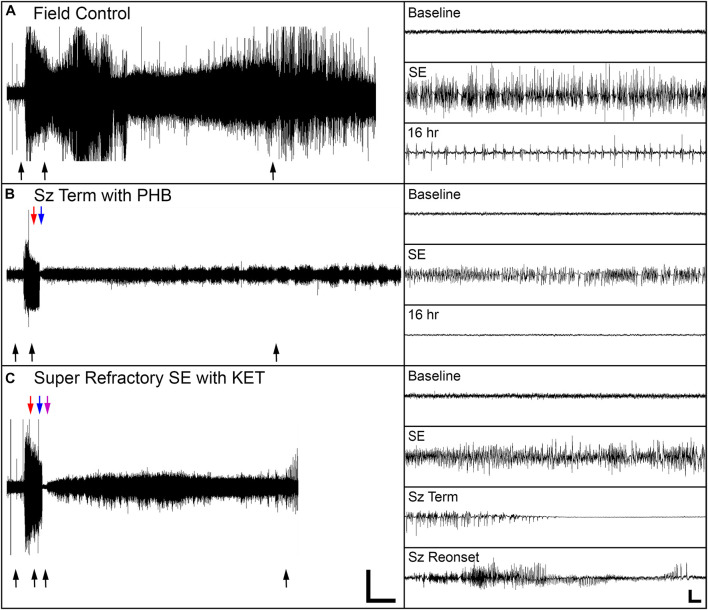
Representative EEG traces from one field control rat and two rats that were treated in the ICU. **(A)** The field control rat remained in SE for the duration of the 24 h recording session. **(B)** For one ICU-treated rat, SE terminated when phenobarbital (PHB) was delivered, and the rat remained seizure-free for 24 h. **(C)** For the other ICU-treated rat, SE initially terminated with ketamine (KET), but seizure activity returned as KET was withdrawn. Black arrows indicate location of insets on the right side of the panels. Red, blue, and purple arrows indicate treatment times for first-, second-, and third-line drugs, respectively. Scale bars for the main panels show 2 h in the *x*-direction and 0.5 mV in the *y*-direction. Scale bars for the insets shows 5 s in the *x*-direction and 0.25 mV in the *y*-direction.

Seizure control and 24 h survival rates were poor for both the field control and the ICU control groups (Table 1). There was no significant difference between control groups in either measure, suggesting that supportive care alone had little effect on NA casualties. In rats that received pharmacological interventions, LRZ and MDZ were largely unable to control NA-induced SE, and both completely failed to provide seizure control that lasted for 24 h. Despite the inefficacy of these benzodiazepines, survival through this first line of treatment remained high, indicating that complications from these drugs were likely not a significant contributor to mortality in NA-poisoned rats. All rats that survived through first-line benzodiazepine treatment met the criteria for established SE and progressed to treatment with second-line antiepileptic drugs. PHB was significantly better than VPA at initially terminating SE, and though seizures returned in a subset of rats, PHB also provided better lasting seizure control than VPA. This difference emerged after 85 rats had received second-line treatment (*n* = 43 VPA, *n* = 42 PHB), so VPA was eliminated as a treatment option thereafter. The choice of second-line treatment had no effect on the percentage of rats that died during this stage, but significantly more rats died during second-line treatment than during first-line treatment (*p* < 0.0001). This may be attributable to the increased amount of time these rats spent in SE. Rats with persistent or returning seizures after receiving a second-line therapy were categorized as refractory and progressed to third-line anesthetic treatments. By design, both PRO and KET provided complete initial seizure control because they were bolused to effect. In all cases, a single bolus of PRO was sufficient to terminate SE. Three rats that received KET required two boluses, and one rat required three boluses to achieve SE termination. Following SE termination, anesthesia was maintained with continuous drug infusion for 18 h, followed by gradual weaning over 6 h. The majority of rats on third-line treatment died prior to the end of this dosing protocol, with no difference in mortality between the PRO and KET groups. For those that survived, seizure activity returned during continuous infusion or during weaning from the anesthetic agent in all rats except for one that had received KET. EEG traces in Figure 2 show examples of baseline EEG activity, followed by seizure onset (SE), seizure termination, and seizure return in 2C.

**TABLE 1:** Outcomes for each therapeutic regimen.

	Drug	*n*	Initial SE Control	*p*-value	Lasting SE Control	*p*-value	Survival	*p*-value
Control	Field control	28	3 (11%)	0.999	1 (4%)	0.999	9 (32%)	0.739
	ICU control	16	2 (13%)		1 (6%)		4 (25%)	
1^st^ Line	Midazolam	69	7 (10%)	0.536	0 (0%)	0.999	62 (90%)	0.767
	Lorazepam	63	4 (6%)		0 (0%)		58 (92%)	
2^nd^ Line	Phenobarbital	77	43 (56%)	0.036*	15 (19%)	0.001**	48 (62%)	0.154
	Valproate	43	15 (35%)		0 (0%)		33 (77%)	
3^rd^ Line	Propofol	31	31 (100%)	0.999	0 (0%)	0.999	11 (35%)	0.288
	Ketamine	35	35 (100%)		1 (3%)		8 (23%)	

### Short-Term Outcomes

**FIGURE 3:**
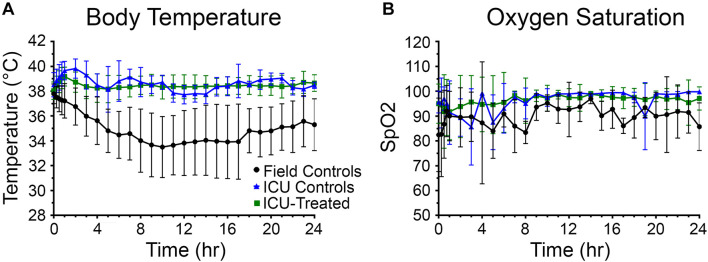
Body temperature and oxygen saturation for rats in each treatment group over 24 h. **(A)** Rats that received thermal support in the ICU environment following nerve agent (NA) exposure were better able to maintain body temperature than field controls. A mixed-effects analysis with Tukey’s multiple comparison test showed that body temperature of field control rats (*n* = 28) differed significantly from ICU controls (*n* = 16) and ICU-treated rats (*n* = 132) at all time points except for 15 min (*p* < 0.05). **(B)** Supplemental oxygen in the ICU environment allowed rats to maintain oxygen saturations > 95% for the majority of the study. Though a mixed-effects analysis with Tukey’s multiple comparison test showed that SpO_2_ levels of field control rats (*n* = 12) did not differ significantly from ICU controls (*n* = 11) or ICU-treated rats (*n* = 80), mean SpO_2_ values for the field control group fluctuated greatly throughout the study. For both panels, data points show means for each treatment group, and error bars indicate standard deviation.

Body temperature data was collected every 15 min for the first hour and then hourly for the next 23 h after entering the ICU environment (*n* = 176). There was a significant effect of time and treatment group on body temperature, and a significant interaction between these variables (*p* < 0.0001 for all). Field control rats had a dramatic reduction in body temperature following NA exposure and were unable to recover to a normal range at any point during the following 24 h. Alternatively, after an initial mild hyperthermic response during the first hour, both treated and control rats that entered the ICU maintained body temperature at 38.6 ± 0.9°C (mean ± SD) (Figure 3A). Significant pairwise differences between field controls and ICU controls were observed at all time points after 15 min, and significant differences between field controls and ICU-treated rats were observed at all time points. Whether or not human NA casualties would require thermal support is debatable, but given that rodents are notoriously poor thermoregulators, supplemental heat is likely an important consideration for preclinical studies (Gordon, 1996; Moffatt et al., 2010; Talaie et al., 2012).

Oxygen saturation (SpO_2_) levels were measured concurrently with body temperatures (*n* = 101). There was no significant effect of time or drug treatment on SpO_2_ levels, suggesting that oxygen saturation was not improved by the supplemental oxygen administered through the ICU environment. However, it is evident in Figure 3B that field control rats were unable to maintain a consistent blood oxygen saturation > 95%, especially during the first 10 h of the study. Meanwhile, rats that were treated in the ICU remained above this benchmark from hour 2 onward. The high variability among the ICU control group is likely due to the small number of rats for which SpO_2_ measurements could be obtained (*n* = 9), and this may be the reason for the observed non-significant result.

### Long-Term Outcomes

**FIGURE 4:**
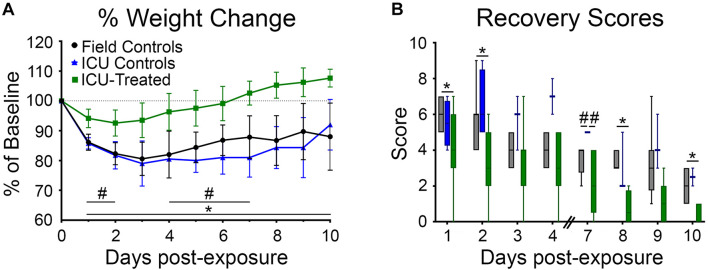
Body weights and recovery scores for rats that were returned to their home cage following NA exposure. **(A)** Body weight was measured daily for 9 days following exposure and reported as percentage of pre-exposure weight. Field controls rats (*n* = 8) never returned to 100% of their initial body weight, and a mixed-effects analysis with Tukey’s multiple comparison test showed that these rats had significantly lower normalized body weights than ICU-treated rats (*n* = 16) on every day post exposure (*p < 0.05). ICU-treated rats also had significantly higher normalized body weights compared to ICU controls (*n* = 4) on post-exposure days 1–2 and 4–7 (#*p* < 0.05). Data points show mean values for each treatment group, and error bars show standard deviation. **(B)** Rats were assessed daily (except on days 5 and 6) for signs of recovery as outlined in Supplementary Table 1, with higher scores indicating poorer condition. A mixed-effects analysis with Tukey’s multiple comparison test showed that ICU-treated rats (*n* = 16) displayed significantly lower recovery scores compared to field controls rats (*n* = 8) on post exposure days 1, 2, 8, and 10. ICU controls had significantly higher recovery scores than both other treatment groups on day 7 post-exposure. Lines show median values for each treatment group, boxes show 25th–75th percentiles, and whiskers show 5th–95th percentiles.

Rats were weighed daily for 10 days following NA exposure because body weight recovery is a key indicator of overall rodent health following poisoning (McDonough et al., 1998). There was a significant effect of time and treatment group on weight change relative to baseline, and a significant interaction between these variables (*p* < 0.0001 for all). Field control and ICU control rats consistently lost weight for the first three days after exposure and did not return to their baseline body weight by the end of the study. For rats that received pharmacological treatment in the ICU, body weight tended to stabilize or begin to recover by 48 h after exposure. All but one treated rat returned to 100% of its baseline body weight by eight days post-exposure. Significant pairwise differences between treatment groups on each day are indicated in Figure 4A.

Additionally, all rats were scored (1–10) on days 1–4 and 7–10 based on body weight loss, breathing, and behavioral reactivity (Figure 4B). A higher score indicated poor recovery and potential for removal from the study based on humane removal criteria (see Supplementary Table 1). There was a significant effect of time and treatment group (*p* < 0.0001 for both) on post-exposure recovery scores, but no interaction between these variables. Scores for all groups decreased over time, validating that this scoring system was a reasonable indicator of recovery. Field controls scored significantly higher than ICU-treated rats, but not ICU controls, at multiple time points. Pairwise differences between groups are shown in Figure 4B. This suggests that pharmacological intervention, not just supportive care, is key to long term recovery following NA-induced SE.

### Behavioral Assays

**FIGURE 5:**
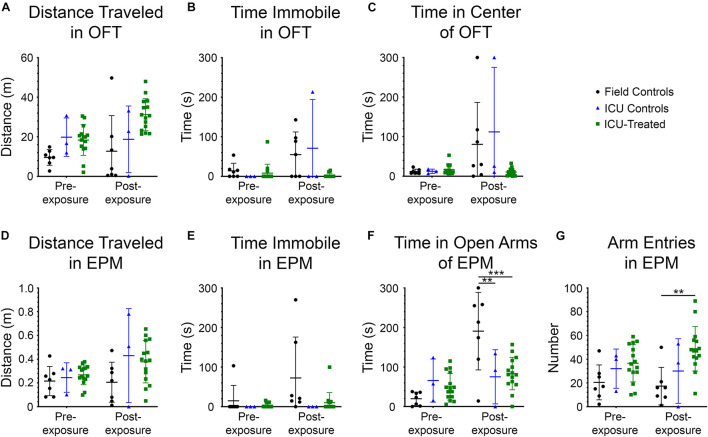
Performance in the open field test (OFT) and elevated plus maze (EPM) for each treatment group before and 9 days after NA exposure. All metrics were evaluated using 2 × 3 way repeated-measures ANOVAs with Sidak’s multiple comparison tests to compare field controls (*n* = 7), ICU controls (*n* = 3), and ICU-treated rats (*n* = 15) at each testing time point. Significant main effects and interactions are reported in the Results section. For the OFT, there were no pairwise differences between treatment groups at either time point for **(A)** total distance traveled, **(B)** time spent immobile, or **(C)** time spent in the center of the field. For the EPM, there were no pairwise differences between treatment groups at either time point for **(D)** distance traveled or **(E)** time spent immobile. **(F)** However, it was observed that there were significant pairwise differences in the amount of time spent in the open arms of the EPM, with field controls spending more time in the open arms than ICU controls and ICU-treated rats post-exposure (**p < 0.01,***p < 0.001). **(G)** Additionally, there were significant pairwise differences in the number of entries into any arm of the EPM, with ICU-treated rats making more entries into arms post-exposure than field controls. This suggests that ICU-treated rats were more exploratory and displayed more typical inhibitory behavior than field controls post-exposure. For all panels, lines show mean values for each treatment group, and error bars show standard deviation.

Rats were tested in the OFT and EPM prior to NA exposure and again at nine days after exposure to evaluate possible effects of NA and subsequent treatments on locomotion, exploration, and anxiety-like behaviors. Measurements collected for the OFT included distance traveled, time immobile, and time in the center of the field (Figures 5A–C). A significant effect of treatment group on distance traveled was observed (*F* (2, 22) = 7.407, *p* = 0.00035), with ICU-treated rats traveling a greater distance than controls. Though there was no interaction between treatment group and time, and pairwise differences between groups were not observed at either time point, this effect appears to be largely driven by ICU-treated rats traveling greater distances during the post-exposure testing session. There was a significant effect of treatment group (*F* (2, 22) = 3.630, *p* = 0.0434), testing time (*F* (1, 22) = 8.127, *p* = 0.0093), and interaction between these variables (*F* (2, 22) = 3.961, *p* = 0.0339) on time spent immobile in the OFT. On average, control rats spent more time immobile during the post-exposure testing session than ICU-treated rats, suggesting that rats that received ICU treatment displayed more consistent exploratory behavior than controls following exposure. A significant effect of time on the amount of time spent in the center of the open field was observed (*F* (1, 22) = 8.707, *p* = 0.0074), with rats spending more time in the center of the field post-exposure than they did before exposure. A significant interaction between treatment group and time was also observed (*F* (2, 22) = 4.018, *p* = 0.0326), with control rats spending significantly more time in the center of the field post-exposure compared to ICU-treated rats. These findings demonstrate that even though thigmotaxis is mildly impaired post-exposure in ICU-treated rats, they show more normal anxiety-related behaviors than controls.

The measurements collected for the EPM included distance traveled, time immobile, time in the open arms of the field, and number of entries into a new arm (Figures 5D–G). No significant effects of testing time, treatment group, or interaction between these variables were observed on distance traveled in this test, confirming that mobility was not impaired following NA exposure. A significant effect of treatment group on the amount of time spent immobile was observed (*F* (2, 22) = 4.595, *p* = 0.0215). Though no interaction between testing time and treatment group was observed, visual inspection of the data suggests that the significant effect of treatment is driven largely by field controls rats spending more time immobile post-exposure, similar to what was observed in the OFT. A significant effect of testing time on time spent in the open arms of the EPM was observed (*F* (1, 22) = 15.45, *p* = 0.0007), with rats spending more time in the open arms at the post-exposure time point. A significant interaction between testing time and treatment group on time spent in the open arms was also observed (*F* (2, 22) = 9.404, *p* = 0.0011) with field control rats spending significantly more time in the open arms post-exposure than both ICU controls and ICU-treated rats. Like the results of the OFT, these findings demonstrate decreased inhibition in control rats after NA exposure that was at least partially mitigated by ICU treatment. Finally, there was a significant effect of treatment group on entries into new arms (*F* (2, 22) = 7.616, *p* = 0.0031). ICU-treated rats demonstrated significantly more arm entries post-exposure than field controls, indicative of heightened exploratory drive.

### Histopathology

**FIGURE 6:**
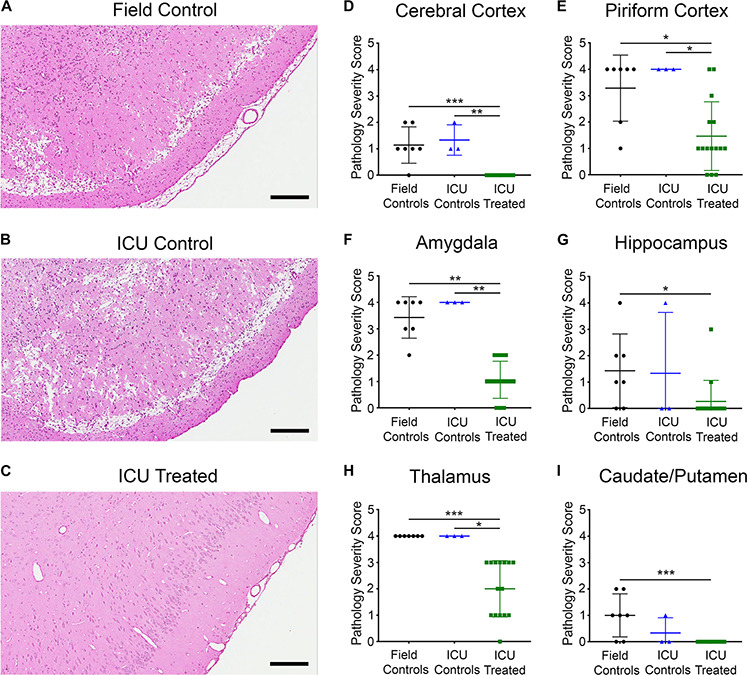
Representative images of H&E staining in the piriform cortex of rats from each treatment group **(A–C)**, and pathology severity scores in six brain regions **(D–I)**. Marked neuronal necrosis and parenchymal loss were evident for both **(A)** the field control rat and **(B)** the ICU control rat, while **(C)** the ICU-treated rat appeared relatively unaffected in the same region. Scale bars for panel **(A–C)** represent 200 μm. Proportion of neurons exhibiting damage in each region from individual field controls (*n* = 7), ICU controls (*n* = 3), and ICU-treated rats (*n* = 15) was scored by a board-certified pathologist according to the following scale: 0 = No lesion; 1 = Minimal (1–10%); 2 = Mild (11–25%); 3 = Moderate (26–45%); and 4 = Severe (> 45%). Regions examined included **(D)** cerebral cortex, **(E)** piriform cortex, **(F)** amygdala, **(G)** hippocampus, **(H)** thalamus, and **(I)** caudate putamen. Within each region, scores for each treatment group were compared using Kruskal–Wallis tests with Dunn’s multiple comparison. Significant pairwise differences are indicated as follows: *p < 0.05, **p < 0.01, ***p < 0.001. For panels **(D–I)**, lines show mean values for each treatment group, and error bars show standard deviation.

Eleven days post exposure, rats were euthanized, and their brains were collected for histopathology analysis. Brain sections underwent H&E staining, and six regions known to be susceptible to NA-induced SE damage were scored by a board-certified pathologist. Brain sections were scored as follows for each region of interest: 0 = no lesion; 1 = minimal (1–10% of neurons exhibiting damage or lost); 2 = mild (11–25%); 3 = moderate (26–45%); and 4 = severe (>45%). Figures 6A–C shows H&E staining in the piriform cortex of a field control, an ICU control, and an ICU-treated rat. The control rats had broad areas of neuronal degeneration and necrosis with significant parenchymal loss, whereas the ICU-treated rats displayed minimal to no signs of neuronal damage. There were significant effects of treatment on pathology scores in all brain regions examined, which included the cerebral cortex (*p* < 0.0001), piriform cortex (*p* = 0.0052), amygdala (*p* = 0.0001), thalamus (*p* < 0.0001), hippocampus (*p* = 0.039), and caudate/putamen (*p* = 0.0041). Rats that received treatment in the ICU had significantly less pathology compared to both control groups in four of the six regions, with pairwise differences displayed in Figures 6D–I. Notably, no damage was observed in any ICU-treated rats for certain brain regions, and the lone rat that received third-line KET had no damage in any region. This illustrates that pharmacological intervention is key in preventing NA-induced neuropathology in this model.

## Discussion

The overarching intent of this study was to determine if current clinical guidelines for the treatment of SE can definitively resolve SE that results specifically from NA exposure. Consistent with numerous other preclinical experiments, benzodiazepines were ineffective even at doses that scale to roughly four times the effective dose levels from the RAMPART study (Jackson et al., 2019; Marrero-Rosado et al., 2018; McDonough et al., 2010; Niquet et al., 2020; Shih et al., 1999; Silbergleit et al., 2012; U.S. Food and Drug Administration (USFDA), 2005). This suggests that NA casualties that do not respond to MDZ in the prehospital environment may be unlikely to benefit from repeated dosing upon hospital arrival and should be considered to be in established SE. Given the depressive effects that benzodiazepines can have on respiration, use of high doses in the NA-poisoned patient could exacerbate already-compromised breathing due to hypersecretions in the lungs, airway constriction, and centrally-driven apnea. This would be best avoided, especially if there is little likelihood of achieving seizure control with these drugs.

Of the two second-line drugs tested, PHB was significantly more effective than VPA at terminating SE, but still only provided lasting relief in 19% of cases. Earlier administration of PHB could improve efficacy since its primary molecular target, GABA_*A*_ receptors, are increasingly internalized as SE progresses (Deeb et al., 2012). This is further reason to avoid iterative benzodiazepine administration, as time spent waiting for benzodiazepines to fail may reduce the efficacy of more promising therapies. PHB and VPA were chosen for comparison because, in addition to their frequent use against benzodiazepine-refractory SE, PHB and derivatives of VPA have previously been shown to terminate organophosphate-induced SE (White et al., 2012; Shekh-Ahmad et al., 2013, 2015; Bar-Klein et al., 2014; Haines et al., 2019; Jackson et al., 2019; Reddy et al., 2020; Spampanato et al., 2020). It is also possible that other common second-line antiseizure drugs like FOS and LEV would provide greater benefit against NA-induced SE. Though the ESET Trial demonstrated equivalent efficacy of VPA, FOS, and LEV as second-line therapies (Kapur et al., 2019), the specific neurochemical milieu of NA-induced SE may respond more readily to a particular drug’s mechanism of action. This will be important to investigate in future studies.

The majority of surviving rats in this study progressed to treatment with KET or PRO. Though initially effective at controlling SE, all but one rat had breakthrough seizures during prolonged continuous dosing or weaning from these drugs. This is the first reported demonstration of SRSE in a rodent model. With further refinement, this model could be instrumental in elucidating the molecular and circuit-based drivers behind development of SRSE. Perhaps even more critically, it could also provide a preclinical tool for screening novel treatment modalities. This is particularly important because there are few therapeutic options for SRSE, and those that are currently employed lack rigorous evidence to support their efficacy. With a mortality rate of 30–50% and severe functional impairments for the majority of survivors (Alvarez and Drislane, 2016), finding better ways to treat SRSE is absolutely imperative.

The survival rate of the rats in this study was poor, particularly on third-line treatment. Though the positive correlation between mortality and progression to RSE is consistent with clinical literature, the cause of death in the human population is typically attributed to severe underlying etiology of the SE. While NA exposure causes systemic toxicity that undoubtedly contributed to mortality throughout the study, additional interventions such as artificial ventilation may have increased survivability. Unfortunately, intubation of a convulsing, NA-exposed rat was not experimentally feasible. Additional responsive interventions may also have been possible if cardiovascular monitoring and blood chemistry data had been available. NA and SE in general lead to time-dependent fluctuations in cardiovascular function, glucose utilization, and metabolism (Howse, 1979; Pazdernik et al., 1985; Young and Koplovitz, 1995; Shorvon, 2001; Hulse et al., 2014). Non-invasive blood pressure cuffs for rodents are available, and the pre-implanted jugular vein catheters that were used to deliver pharmacological interventions could also be used to take blood samples for rapid analysis on a handheld device. These systems could allow for strategic administration of pressors, sodium bicarbonate, i.v. fluids, and similar supportive therapies in future work with this model.

Importantly, this study demonstrated that functional recovery from NA-induced SE is possible when pharmacological seizure control is achieved. Control rats demonstrated behavioral deficits and severe pathology that were consistent with previous reports (McDonough et al., 1986, 1995, 1998; Raffaele et al., 1987; Guignet et al., 2020). The most marked behavioral alteration in control animals after NA exposure was their lack of species-typical caution and inhibition. In addition to spending large amounts of time in the center of the OFT and the open arms of the EPM, several rats repeatedly and purposefully jumped off the arms of the EPM (see Supplementary Video 1). Though some degree of brain damage did occur in several key regions for the rats that were successfully treated in this study, their performance in the behavioral assays was more similar to that of baseline. This suggests that even if seizure control takes a long time to achieve, meaningful recovery of function and preservation of quality of life could be possible. The prognosis for NA-induced RSE patients may be more promising than that for the general RSE population because advanced age and pre-existing structural abnormalities, which are highly correlated with poorer outcomes, are less likely to be complicating factors (Alvarez and Drislane, 2016).

Because treatment protocols for NA exposure cannot be tested in clinical trials, reliance on animal models to guide therapeutic decisions is necessary. There is, of course, no way to know how well the results from this rat study would translate to a human NA casualty, but it does provide a framework where none has existed thus far. It is clear that the SE produced in this model is particularly severe, with a large proportion of subjects entering SRSE. Interrogation of SRSE in a manipulable animal model could help the scientific and medical community begin to understand this dire state for which little evidence-based recourse currently exists. Continued studies will focus on refining the fidelity of the simulated clinical environment, exploring how the timing and choice of second-line therapies alters outcomes, and beginning to dissect the origins of SRSE emergence.

## Data Availability Statement

The raw data supporting the conclusions of this article will be made available by the authors, without undue reservation.

## Ethics Statement

This study was approved by the Institutional Animal Care and Use Committee (IACUC) at the United States Army Medical Research Institute of Chemical Defense, and all procedures were conducted in accordance with the principles stated in the Guide for the Care and Use of Laboratory Animals, and the Animal Welfare Act of 1966 (P.L. 84-544).

## Author Contributions

HSM designed this research study. JEM, SCW, BJT, KHB, KTP, HMB, CJ, KMB, JMC, ENH, and HSM conducted the experiments, acquired the data, and provided daily care to study animals. JM, SW, KHB, and HSM analyzed the data. JEJ evaluated and scored the histopathological samples. JEM, SCW, BJT, KHB, and HSM wrote the manuscript.

## Conflict of Interest

The authors declare that the research was conducted in the absence of any commercial or financial relationships that could be construed as a potential conflict of interest.

## Publisher’s Note

All claims expressed in this article are solely those of the authors and do not necessarily represent those of their affiliated organizations, or those of the publisher, the editors and the reviewers. Any product that may be evaluated in this article, or claim that may be made by its manufacturer, is not guaranteed or endorsed by the publisher.
