# Supplementary material for: Refractory and Super-Refractory Status Epilepticus in Nerve Agent-Poisoned Rats Following Application of Standard Clinical Treatment Guidelines
Source: Front Neurosci. 2021 Sep 10;15:732213. doi: 10.3389/fnins.2021.732213 (PMC8462486; doi:10.3389/fnins.2021.732213)
Supplement: Supplementary file 1 [file Table_1.DOCX]

Supplementary Table 1: Post-exposure recovery scoring guide. In addition to serving as a metric of recovery over time, scores of 6+ qualified rats for humane removal from the study unless mitigating circumstances were identified by the principal investigator and attending veterinarian.

| Score | 0 | 1 | 2 | 3 |
| --- | --- | --- | --- | --- |
| % Change from pre-exposure weight | <10% | 10-25% | 26-30% | >30% |
| Breathing | Normal | Mild to moderate abdominal breathing | Moderated to marked abdominal breathing w/ cyanosis | Foamy and/or bloody discharge |
| Natural Behavior | Normal | Slight lethargy, alert | Moderate lethargy or restlessness, alert | Extreme lethargy or restlessness, no alert |
| Provoked Behavior | Normal | Minor depression or exaggeration of behavior | Moderate change in behavior, unusual aggression or blunted response | Unresponsive to external stimulus |
