# Supplementary material for: Refractory and Super-Refractory Status Epilepticus in Nerve Agent-Poisoned Rats Following Application of Standard Clinical Treatment Guidelines
Source: Front Neurosci. 2021 Sep 10;15:732213. doi: 10.3389/fnins.2021.732213 (PMC8462486; doi:10.3389/fnins.2021.732213)
Supplement: Supplementary file 2 [file Table_2.DOCX]

Supplementary Table 2: Comparison of long-term outcome metrics for rats treated with MDZ + PHB and LRZ + PHB. No significant differences were observed between these groups for any metric, so data from both groups were combined into a single “ICU-Treated” group for comparison to controls.

| Metric | P Value | Statistical Test |
| --- | --- | --- |
| Body Weight | 0.8008 | Mixed-effects analysis  *(p value for effect of treatment group reported)* |
| Recovery Score | 0.9877 |  |
| OFT- Distance Traveled | 0.1527 | Unpaired t-test  *(post-exposure data only)* |
| OFT- Time Immobile | 0.3283 |  |
| OFT- Time in Center | 0.1385 |  |
| EPM- Distance Traveled | 0.1932 |  |
| EPM- Time Immobile | 0.3379 |  |
| EPM- Time in Open Arms | 0.0810 |  |
| EPM- Number of Arm Entries | 0.6310 |  |
| Histopathology- Cerebral Cortex | >0.9999 | Mann-Whitney test |
| Histopathology- Piriform Cortex | 0.3433 |  |
| Histopathology- Amygdala | 0.6630 |  |
| Histopathology- Thalamus | >0.9999 |  |
| Histopathology- Hippocampus | 0.4725 |  |
| Histopathology- Caudate/Putamen | >0.9999 |  |
