# Supplementary material for: Refractory and Super-Refractory Status Epilepticus in Nerve Agent-Poisoned Rats Following Application of Standard Clinical Treatment Guidelines
Source: Front Neurosci. 2021 Sep 10;15:732213. doi: 10.3389/fnins.2021.732213 (PMC8462486; doi:10.3389/fnins.2021.732213)
Supplement: Supplementary file 4 [file Data_sheet_1.DOCX]

Supplementary Video 1: A field control rat in the elevated plus maze during pre-exposure and post-exposure testing. The video is presented at 2x speed. During pre-exposure testing, the rat spends the majority of its time in the closed arms of the maze, with brief explorations of other arms. During post-exposure testing, the rat spends far more time in the open arms of the maze and repeatedly jumps off the maze, demonstrating a marked lack of inhibition.
